# Toxicity of insulin-derived amyloidosis: a case report

**DOI:** 10.1186/s12902-019-0385-0

**Published:** 2019-06-13

**Authors:** Keiichi Iwaya, Tamotsu Zako, Junta Fukunaga, Karin Margareta Sörgjerd, Kentaro Ogata, Koichiro Kogure, Hiroshi Kosano, Masayuki Noritake, Mizuo Maeda, Yukio Ando, Yoshiya Katsura, Terumasa Nagase

**Affiliations:** 1 Department of Pathology, SASAKI Institute, Kyoundo Hospital, Tokyo, Japan; 2 Bioengineering Laboratory, RIKEN Cluster for Pioneering Research, Saitama, Japan; 3 Department of Chemistry and Biology, Graduate School of Science and Engineering, Ehime University, Ehime, Japan; 4 Department of Pathology, Tachikawa Hospital, Tokyo, Japan; 5 Department of Metabolism and Endocrinology, Tokyo Medical University Ibaraki Medical Center, 3-20-1 Chuou, Ami, Ibaraki, 300-0395 Japan; 6 Faculty of Pharmaceutical Science, Teikyo University, Tokyo, Japan; 7 Department of Neurology, Graduate School of Medical Sciences, Kumamoto University, Kumamoto, Japan

**Keywords:** Insulin, Amyloid, Insulin ball, Fibril, Filament, Non-toxic, Necrosis, Minocycline

## Abstract

**Background:**

Insulin-derived amyloidosis is a skin-related complication of insulin therapy that interferes with insulin therapy. Although toxicities of in vitro-formed insulin amyloid fibrils have been well studied, the toxicity of insulin-derived amyloidosis remains to be clarified.

**Case presentation:**

A 58-year-old man with type 2 diabetes mellitus underwent a lower limb amputation due to diabetic gangrene. Several antibiotics including minocycline were administered for infection and sepsis. A hard mass at the insulin injection sites in the lower abdomen was discovered by chance four months later. Although no abnormal findings in the surface skin of the mass were observed, necrotic tissue was seen around the mass when a biopsy was performed. Histological and toxicity studies were performed for this patient and four other patients with abdominal masses at insulin injection sites. Histological and immunohistochemical studies showed that the masses had typical characteristics of amyloid deposits in all cases, whereas necrotic findings were seen adjacent to the amyloid deposit only in the case presented. Toxicity studies indicated that the amyloid tissue from the present case had significant cell toxicity compared to the control skin tissue or the amyloid tissues from the other four cases.

**Conclusions:**

This report showed that toxic insulin-derived amyloidosis can occur. In addition, this report suggested that toxic insulin-derived amyloidosis may cause necrosis in the surrounding tissue. Although the toxic amyloid deposit of insulin-derived amyloidosis was found in only one patient, no structural differences between toxic and non-toxic deposits were seen on histological and immunohistochemical studies.

**Electronic supplementary material:**

The online version of this article (10.1186/s12902-019-0385-0) contains supplementary material, which is available to authorized users.

## Background

Insulin-derived amyloidosis is a skin-related complication of insulin therapy. The amyloid fibril protein is derived from the injected insulin and forms an amyloid deposit at the injection site [1–7]. Our previous studies showed that insulin-derived amyloidosis causes poor glycemic control and increased insulin dose requirements because of impairments in insulin absorption [3, 5]. Thus, the existence of insulin-derived amyloidosis interferes with insulin therapy. However, the toxicity of insulin-derived amyloidosis itself remains to be clarified.

In vitro formation of insulin amyloid fibrils has been well studied [8, 9], and toxicity of insulin amyloid fibrils has also been investigated [10–12]. In some other amyloidogenic proteins, oligomeric intermediates have been shown to be more toxic than mature amyloid fibrils, and the toxicity of oligomeric intermediates is considered to be associated with the pathogenesis of amyloid-related diseases such as Alzheimer’s disease [13]. However, as for insulin, the relationship between toxicity and species of insulin amyloid fibrils remains controversial [10, 11]. Additionally, our group recently showed that there are two different forms of mature insulin amyloid: toxic insulin fibrillar amyloid (insulin fibrils) and non-toxic insulin filamentous amyloid (insulin filaments) [14, 15].

In this report, a case of toxic insulin-derived amyloidosis in which the effect of toxicity on the surrounding tissue was examined is presented. In addition, the relationship between the toxicity and the use of antibiotics is considered.

### Case presentation

A 58-year-old Japanese man with a 17-year history of type 2 diabetes mellitus was admitted to our hospital because of diabetic gangrene of his left lower limb. This case has been briefly described previously [5]. A transfemoral amputation was performed due to widespread infection, and blood culture showed *Staphylococcus haemolyticus* sepsis. Therefore, the following antibiotics were administered before and after the amputation: minocycline 100 mg/day intravenously for one week and orally for two weeks; ceftriaxone 1 g/day intravenously for 11 days; vancomycin 1 g/day intravenously for one week; and clindamycin 1800 mg/day intravenously for one week. The doses of the antibiotics were decreased because his renal function was severely impaired (serum creatinine level, 5.02 mg/dL).

After acute-phase treatment was finished, he continued to be hospitalized for rehabilitation with an artificial leg. About four months after the admission, a hard mass at the insulin injection sites in the left lower abdomen was discovered by chance. He had almost always injected insulin at those sites before admission, but he mainly injected insulin at other sites in the abdomen after admission. At the time that the mass was discovered, he had no fever and no pain, and the laboratory tests showed no signs of inflammation (white blood cell count, 7.30 × 10^3^/μL; serum C-reactive protein level, 0.01 mg/dL). In addition, no abnormal findings of the surface skin of the mass were observed. When skin incision biopsy of the mass was performed, necrotic tissue was seen around the mass. Therefore, a drainage tube was put into the necrotic tissue for two days, an empirical oral antibiotic was administered for four days, and the wound healed in two weeks.

### Tissue samples and participants

Tissue samples for subsequent studies were prepared from five patients, Cases 1–5, who had abdominal masses at their insulin injection sites (Table 1). The case presented above is Case 5. The clinical characteristics of these cases have been described previously [5].

**Table 1:** Clinical characteristics of study participants

Case no.	Age (y)	Sex	Insulin duration (y)	Insulin preparation	Insulin dose (U/d)
1	62	Male	20	Lispro + Glargine	116
2	82	Female	12	Lispro + Glargine	30
3	71	Male	25	Lispro + Glargine	47
4	68	Male	18	Lispro + Glargine	33
5	58	Male	15	Lispro + Glargine	52

Tissue samples were obtained by skin incision biopsy of an abdominal mass in five patients. Each of the samples was trimmed into two pieces. One was processed into a formalin-fixed, paraffin-embedded block for histological and immunohistochemical studies. The other piece was processed into a frozen tissue block for toxicity studies.

### Histological and immunohistochemical studies

Tissue specimens that were cut from paraffin-embedded blocks were stained with hematoxylin-eosin and Congo red and immunostained with anti-insulin mouse monoclonal antibodies (IN-05, Monosan, Uden, The Netherlands) and anti-serum amyloid P component (SAP) monoclonal antibodies (Novocastra, Newcastle-upon-Tyne, UK).

**Fig. 1:**
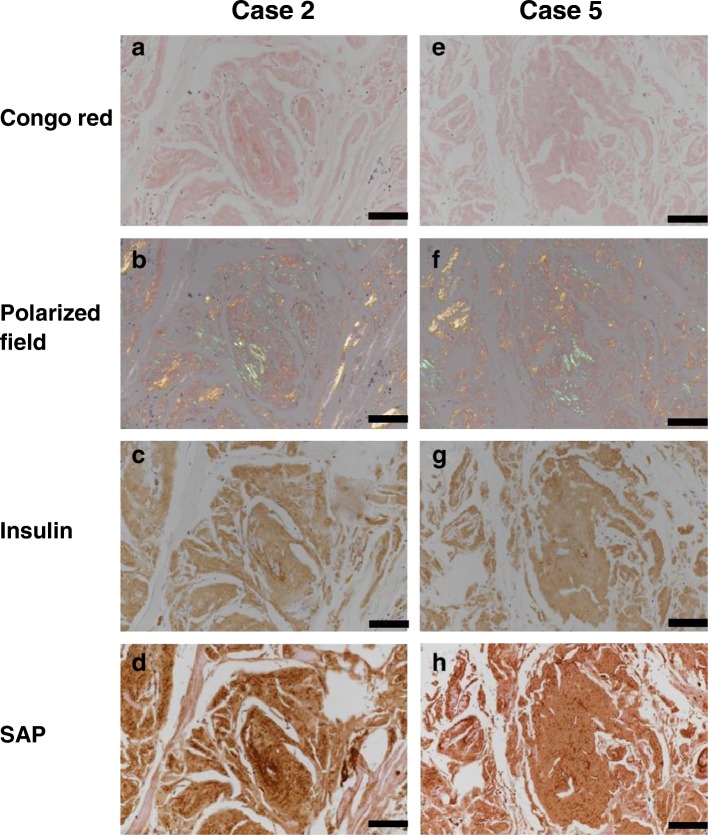
Histological findings of the abdominal masses at the insulin injection sites in two cases (Cases 2 and 5). A figure set (**a**, **b**, **c,** and **d**) was obtained from Case 2, and another set (**e**, **f**, **g,** and **h**) was obtained from Case 5. The masses are stained with Congo red (**a** and **e**) and viewed under a crossed polarized field (**b** and **f**). Insulin is detected using immunostaining with anti-insulin antibody (**c** and **g**). Serum amyloid P component (SAP) is detected in almost the same area immunoreacted with insulin (**d** and **h**). Original magnification, 400 x; bar, 100 μm

All biopsied masses corresponded histologically to the amorphous deposits that were positive for apple-green birefringence by Congo red staining and for insulin by immunostaining (Fig. 1), confirming that the masses were insulin-derived amyloidosis [5]. In addition, the deposits were positive for SAP by immunostaining. These histological findings were similar across all cases (representative cases, Case 2 and Case 5, Fig. 1).

**Fig. 2:**
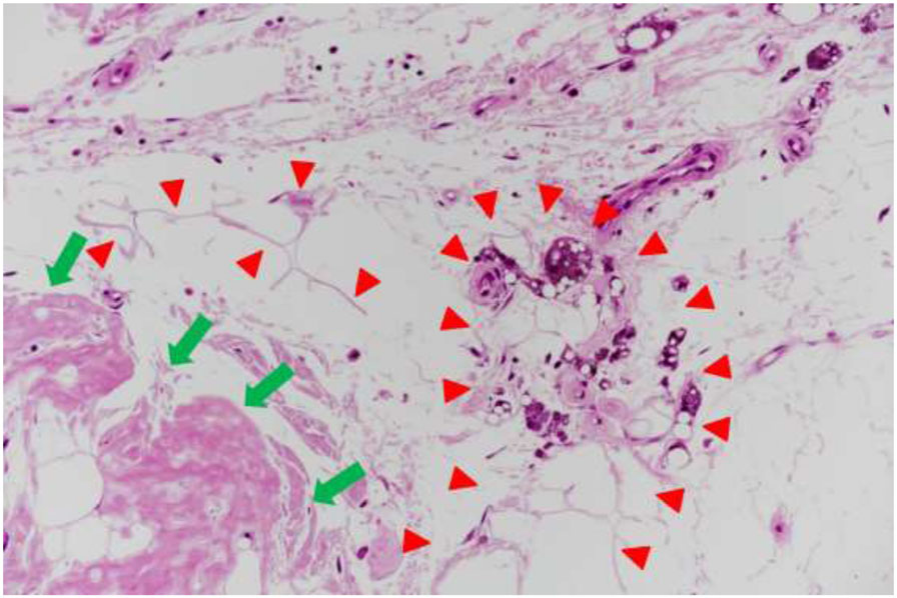
Fat necrosis in the surrounding tissue of the amyloid deposit in Case 5. Distortion of the septa of fat tissue and foamy cells (indicated by red arrowheads) are seen in the subcutaneous fat tissue adjacent to the amyloid deposit (green arrows). Hematoxylin-eosin staining; original magnification, 100 x

Although the surface skin of the mass was normal in all cases, necrotic tissue was seen around the mass on biopsy in Case 5. Light microscopic evaluation of the sample from Case 5 showed necrotic findings, namely distortion of the septa of fat tissue and foamy cells in the subcutaneous fat tissue adjacent to the amyloid deposit (Fig. 2), and many macrophages were present around the necrotic tissue. In contrast, no necrotic findings were observed on light microscopic examination of the samples from the four other cases.

### Toxicity studies

The frozen tissue block was cut into 10-μm-thick specimens, and Congo red staining was performed for every five slices. The area of insulin-derived amyloidosis that corresponded to the Congo red-positive portion on each specimen was microdissected. Other areas of tissue, including the epidermis, dermis, and subcutaneous tissue, were also microdissected as a control; thus, the control was pooled tissue from Cases 1–5 because the amount of tissue from one case was small. The powdered tissue was suspended in sterilized phosphate-buffered saline (PBS).

Cell toxicity assays were performed using a water-soluble tetrazolium salts-8 assay (Dojindo, Kumamoto, Japan) [14]. Each of the powdered tissue samples (re-suspended 12 μg of powdered tissue up to 10 μL of PBS (W/V)) was applied for 24 h to two different human cell lines, HEK293T (1.25 × 10^3^/100 μL) and HeLa cells (6.25 × 10^4^/100 μL), pre-cultured in each well. Cell viability was evaluated by comparing the number of viable cells after the addition of powdered tissue samples with 100% viable cells after the addition of PBS alone. The assays were performed twice using triplicate samples in each experiment. The difference among the mean values of cell viability was assessed by one-way analysis of variance (ANOVA). After a significant difference was obtained by one-way ANOVA, the post hoc Tukey test was performed. A *p* value < 0.05 was considered significant.

**Fig. 3:**
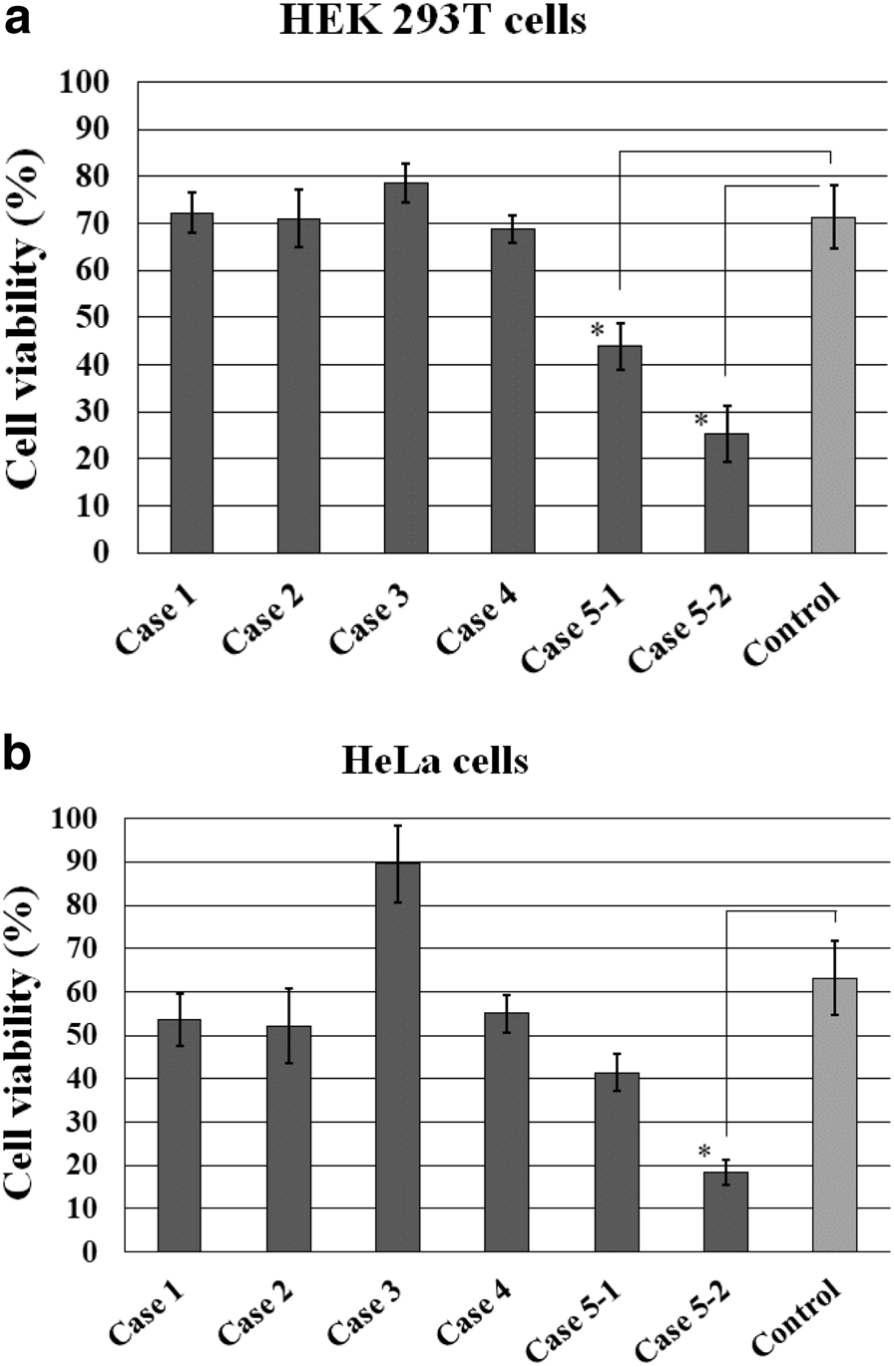
Toxicity of insulin-derived amyloidosis in Cases 1–5. Two samples separately prepared from Case 5 (Case 5–1 and 5–2) in the HEK293T cell assay (**a**) and one sample (Case 5–2) in the HeLa cell assay (**b**) show significantly lower cell viability than the control. Error bars, SE; *, *p* < 0.05

The toxicities of the tissue samples against HEK293T cells and HeLa cells are shown in Fig. 3. Two samples separately prepared from Case 5 (Case 5–1 and 5–2) in the HEK293T cell assay (Fig. 3) and one sample (Case 5–2) in the HeLa cell assay (Fig. 3b) showed significantly lower cell viability compared with the control or the other four samples from Cases 1–4, indicating that they had significant cell toxicity. In contrast, no significant differences in cell viability were found between the four other samples and the control in both assays.

## Discussion and conclusion

This is the first case of toxic insulin-derived amyloidosis of the over 80 previously reported cases [1–7]. The amyloid tissue of the present case showed significant toxicities against both HEK293T cells and HeLa cells compared to the control skin tissue or the amyloid tissues of the other four cases. Although toxicities of in vitro-formed insulin amyloid fibrils have been well studied [10–12, 14, 15], the toxicity of in vivo-formed insulin-derived amyloidosis has not been reported. Therefore, this is an important and valuable case showing that insulin-derived amyloidosis could be toxic to cells in some instances.

In addition, this is the only case with necrotic tissues around the amyloid deposit of the seven reported patients [5]. Since systemic and local inflammatory reactions were not observed in this case at the time that the mass was discovered, it was unlikely that necrosis was induced by infections. In fact, bacterial and fungal infections were not observed on the histological studies. Additionally, other causes of necrosis, such as peripheral vascular disorders or vasculitis, were not observed on histological examination. Since toxic species of amyloid proteins have been shown to cause cell death through disassembling and permeabilizing the cell membrane [16, 17], we presume that the toxic insulin-derived amyloidosis may cause necrosis.

The histological examinations of Congo red staining and SAP immunostaining indicated that both toxic and non-toxic deposits have the same typical characteristics of amyloid deposits [18], as shown in Fig. 1. Therefore, there were no structural differences between the toxic and non-toxic deposits on the histological and immunohistochemical examinations. According to the in vitro studies, mature insulin amyloid fibrils generally have toxicities [10–12, 14]. In contrast, the present study showed that the amyloid deposits of insulin-derived amyloidosis do not have toxicities in most cases. The cause of this discrepancy between in vitro-formed insulin amyloid fibrils and in vivo-formed amyloid deposits is not known. One possibility is that the non-toxic amyloid deposits of insulin-derived amyloidosis consist of insulin filaments, but not insulin fibrils [14]. Since the in vitro-formed insulin filaments are non-toxic [14, 15], it is plausible that the amyloid deposits are also non-toxic if they consist of insulin filaments. However, further studies, including electron microscopy, are necessary to clarify the relationship between toxicity and the structure of insulin-derived amyloidosis.

The present case with toxic insulin-derived amyloidosis had a history of diabetic gangrene with sepsis and had taken antibiotics including minocycline for several weeks before the amyloidosis was found. Such conditions, especially the use of antibiotics, may have caused a structural change in the amyloid deposits of insulin-derived amyloidosis, which may be involved in inducing toxicity. In fact, minocycline might be a possible cause for the structural change, because tetracycline and its derivatives have been reported to disrupt amyloid fibrils of transthyretin [19], β2-microglobulin [20], and immunoglobulin light chain [21]. To evaluate the effects of minocycline, incubation studies of minocycline were performed, using two different forms of mature insulin amyloid, insulin fibrils and insulin filaments [14, 15]. The study showed a decrease in the fluorescence of an amyloid probe dye, Thioflavin T, with incubation with pharmacological concentrations (1–5 μM) of minocycline and both forms of insulin amyloid, suggesting a possible structural change of insulin amyloid (Additional file 1: Figure S1). However, further studies are needed to investigate the possible effects of minocycline on insulin-derived amyloidosis.

The clinical characteristics of the present case with toxic insulin-derived amyloidosis were compared with those of the other 15 cases, including the four cases in the present study, with insulin-derived amyloidosis that we had seen. The present case with toxic amyloidosis had type 2 diabetes, whereas six had type 1 diabetes and nine had type 2 diabetes of the 15 cases. The duration of insulin therapy and daily insulin dose in the present case were 15 years and 52 units, respectively, whereas the average duration of insulin therapy and daily insulin dose in the 15 cases were 17 years and 53 units, respectively. Additionally, insulin preparations used in the present case were insulin lispro and insulin glargine (Table 1), and eight of the 15 patients also used insulin lispro and insulin glargine. Thus, the clinical characteristics of the present case with toxic insulin-derived amyloidosis were similar to those of other cases with insulin-derived amyloidosis.

In conclusion, this report showed that toxic insulin-derived amyloidosis can occur. In addition, this report suggested that toxic insulin-derived amyloidosis may cause necrosis in the surrounding tissue. Although the toxic amyloid deposit of insulin-derived amyloidosis was found in only one patient, no structural differences between toxic and non-toxic deposits were found on histological and immunohistochemical studies. Further studies are necessary to clarify the relationship between toxicity and the structure of insulin-derived amyloidosis and to understand the mechanisms that induce the toxicity of insulin-derived amyloidosis.

## Additional file

Additional file 1: **Figure S1**: Effects of minocycline on two types of insulin amyloid (PPTX 52 kb)

## Data Availability

All data generated or analyzed during this study are included in this published article.

## Abbreviations

ANOVA: Analysis of variance
PBS: Phosphate-buffered saline
SAP: Serum amyloid P component
