# Supplementary material for: Toxicity of insulin-derived amyloidosis: a case report
Source: BMC Endocr Disord. 2019 Jun 13;19:61. doi: 10.1186/s12902-019-0385-0 (PMC6567432; doi:10.1186/s12902-019-0385-0)
Supplement: Supplementary file 1 — Figure S1: Effects of minocycline on two types of insulin amyloid (PPTX 52 kb) [file 12902_2019_385_MOESM1_ESM.pptx]

## Slide 1
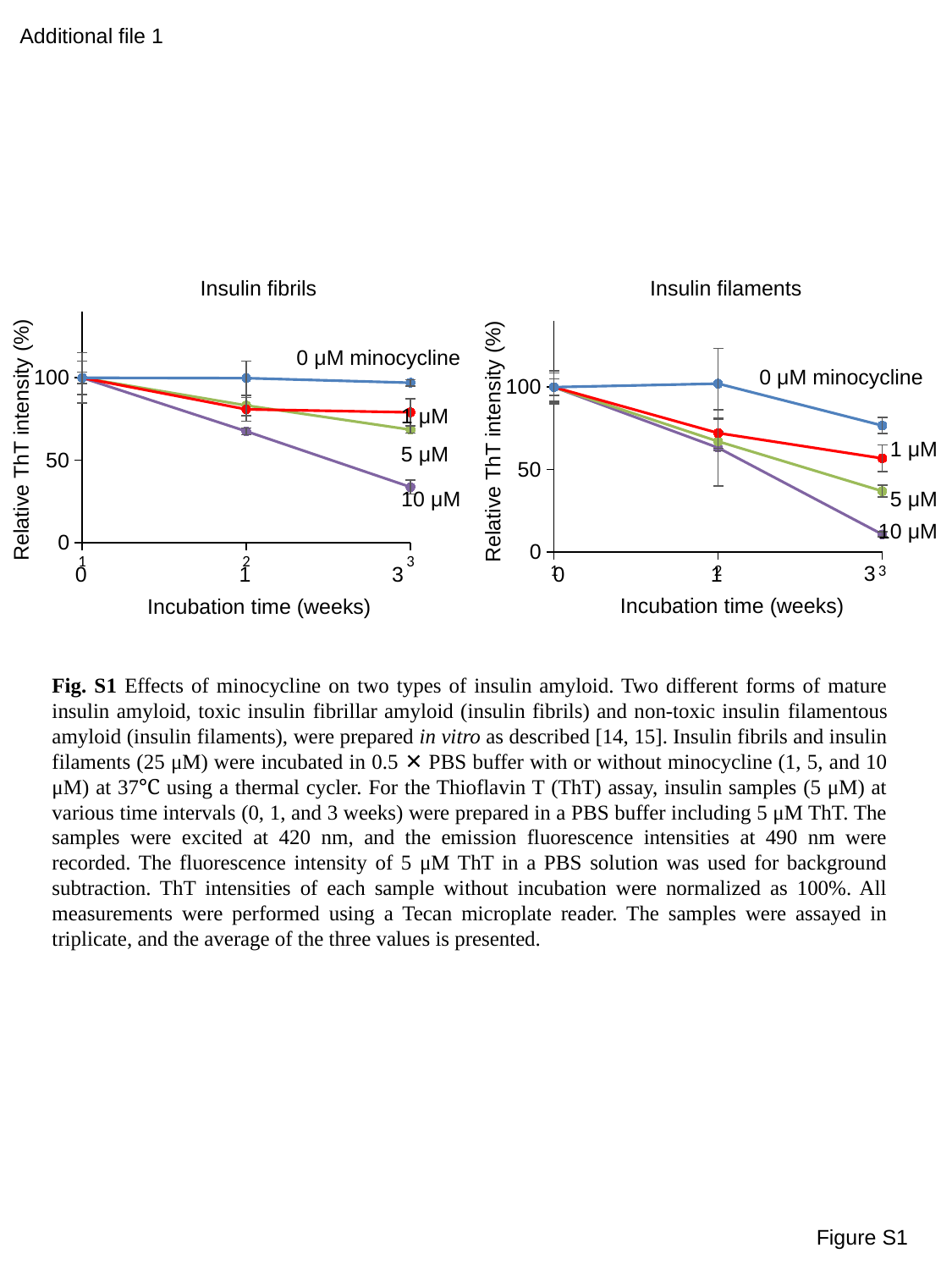

Additional file 1
Insulin fibrils
Insulin filaments
### Chart
| Category | Fib | Fib+1uM MINO | Fib+5uM MINO | Fib+10uM MINO |
|---|---|---|---|---|
### Chart
| Category | Fila | Fila+1uM MINO | Fila+5uM MINO | Fila+10uM MINO |
|---|---|---|---|---|0 μM minocycline
0 μM minocycline
1 μM
Relative ThT intensity (%)
Relative ThT intensity (%)
1 μM
5 μM
10 μM
5 μM
10 μM
3
1
0
1
0
3
Incubation time (weeks)
Incubation time (weeks)
Fig. S1 Effects of minocycline on two types of insulin amyloid. Two different forms of mature insulin amyloid, toxic insulin fibrillar amyloid (insulin fibrils) and non-toxic insulin filamentous amyloid (insulin filaments), were prepared in vitro as described [14, 15]. Insulin fibrils and insulin filaments (25 μM) were incubated in 0.5 ✕ PBS buffer with or without minocycline (1, 5, and 10 μM) at 37℃ using a thermal cycler. For the Thioflavin T (ThT) assay, insulin samples (5 μM) at various time intervals (0, 1, and 3 weeks) were prepared in a PBS buffer including 5 μM ThT. The samples were excited at 420 nm, and the emission fluorescence intensities at 490 nm were recorded. The fluorescence intensity of 5 μM ThT in a PBS solution was used for background subtraction. ThT intensities of each sample without incubation were normalized as 100%. All measurements were performed using a Tecan microplate reader. The samples were assayed in triplicate, and the average of the three values is presented.
Figure S1
